# Enamel remineralization potential of bioactive glass-containing toothpastes: an in vitro study

**DOI:** 10.1007/s10266-026-01337-z

**Published:** 2026-02-09

**Authors:** Pakize Rana Çetin, Elif Alkan, Dilek Tağtekin, Ali İhsan Çetin, Nihal Şehkar Oktay

**Affiliations:** 1 Department of Restorative Dentistry, Faculty of Dentistry, Marmara University, Recep Tayyip Erdoğan Complex Health Campus, Basibuyuk Road No. 9/3, Basibuyuk, Maltepe, 34854 Istanbul, Turkey; 2 Faculty of Business Administration, Ankara Yıldırım Beyazıt University, Esenboğa Campus, Block B, 2nd Floor, Dumlupınar District, Esenboğa, Çubuk, Ankara, Turkey; 3 Division of Basic Medical Sciences, Department of Basic Medical Sciences in Dentistry, Faculty of Dentistry, Marmara University, Recep Tayyip Erdoğan Complex Health Campus, Basibuyuk Road No. 9/3, Basibuyuk, Maltepe, 34854 Istanbul, Turkey

**Keywords:** Enamel remineralization, Bioactive glass, Hydroxyapatite, Fluoride, Toothpaste

## Abstract

This study evaluated the remineralization potential of experimental containing bioactive glass, hydroxyapatite, protein–calcium glycerophosphate, and their fluoride-enriched formulations on artificially demineralized enamel. Eighty extracted human molars were sectioned into enamel specimens, and artificial caries lesions were induced. Specimens were randomly divided into eight groups (*n* = 10): experimental formulations (bioactive glass (MB), bioactive glass + fluoride (MBF), hydroxyapatite (MH), hydroxyapatite + fluoride (MHF), protein + calcium glycerophosphate (MP)), commercial toothpastes (Sensodyne Repair & Protect with NovaMin (MS), R.O.C.S. Sensitive (MR)), and a control group. All specimens underwent a 14-day pH-cycling protocol simulating daily demineralization and remineralization. Remineralization efficacy was assessed at baseline, post-demineralization, and post-treatment using DIAGNOdent Pen, FluoreCam imaging, and Vickers microhardness testing. Data were analyzed using ANOVA. Significance level was set at *p* < 0.05. The highest microhardness recovery occurred in MHF (60.65 ± 21.92) and MS (57.07 ± 24.14), while the least increase was observed in the control (9.46 ± 14.28) and MP (15.5 ± 17.23) (*p* < 0.001). FluoreCam analysis revealed the greatest mineral gain in MHF (4.22 ± 2.24), MS (4.12 ± 1.57), and MBF (4.02 ± 2.73); however, no significance was detected between treatment groups (*p* = 0.419). DIAGNOdent Pen values were lowest post-treatment in MR (6.7 ± 0.9), MBF (6.9 ± 0.94), and MS (6.9 ± 1.14), while all treatment groups had significantly lower values than the control (11.1 ± 1.45) (*p* < 0.001). Experimental toothpastes containing bioactive glass, hydroxyapatite, and protein– calcium glycerophosphate, as well as commercial products containing NovaMin and hydroxyapatite, supported enamel remineralization. The addition of fluoride enhanced this effect. *Clinical significance* This study demonstrates the potential of various remineralization agents to strengthen early enamel lesions, offering clinicians alternative non-invasive options for early caries management.

## Introduction

Dental caries is among the most prevalent chronic diseases globally and develops through complex interactions between the tooth surface, biofilm, diet, saliva, and genetic factors [1]. Enamel is continuously subjected to cycles of demineralization and remineralization in the oral environment [2]. Demineralization occurs when the oral pH drops, leading to the loss of calcium and phosphate ions, while remineralization begins when these ions are redeposited onto the enamel surface [3]. Hydroxyapatite crystals constitute approximately 90–92% of the enamel structure and are particularly susceptible to dissolution in acidic environments [4]. Fluoride’s well-established anticariogenic action includes promoting remineralization, inhibiting demineralization, and exerting antibacterial effects against cariogenic bacteria such as *Streptococcus mutans* [5–7].

White spot lesions are defined as reversible demineralized areas on the enamel surface and can be reversed through remineralization therapies [8]. The effectiveness of topical agents used in this process could be evaluated using microhardness measurements and optical assessment methods [9, 10].

Bioactive glasses, upon contact with saliva, rapidly release ions through their nanosized particles, leading to the release of calcium, phosphorus, and sodium. In recent years, due to their biocompatibility, they have increasingly found applications in dentistry. As a result of a series of reactions in an aqueous environment, a surface layer composed of hydroxyapatite and/or hydroxycarbonate apatite is formed [11]. Compared with other bioactive materials such as hydroxyapatite, their higher bioactivity index enables the rapid formation of a chemical bond with dental tissue, thereby facilitating the remineralization of initial caries lesions [12–14].

Besides bioactive glass, alternative remineralizing agents such as casein phosphopeptides-amorphous calcium phosphate (CPP–ACP), calcium glycerophosphate (CaGP), and synthetic hydroxyapatite have been investigated. These agents contribute to enamel repair by enhancing ion availability and supporting crystal lattice recovery [15–19].

Enamel demineralization is a common early indicator of dental caries observed in both children and adults worldwide. Epidemiological studies have shown that the prevalence of white spot lesions can reach up to 97% in individuals undergoing fixed orthodontic treatment [20]. Although previous studies have investigated various remineralizing agents, there is limited data in the literature regarding the combined use of bioactive glass with fluoride, its particle size characteristics, and its comparison with alternative agents. This study aimed to evaluate the remineralization potential of experimental and commercial toothpastes containing different active agents on artificially demineralized enamel surfaces. The hypotheses of the study were as follows:

1. Experimental toothpastes containing bioactive glass, hydroxyapatite, and protein–calcium glycerophosphate support enamel remineralization.
2. The combination of bioactive glass and hydroxyapatite with fluoride enhances enamel remineralization.

## Materials and methods

### Selection and preparation of tooth specimens

A total of 80 sound, caries-free, and restoration-free human molars extracted for orthodontic reasons within the past 6 months were included in the study, with informed consent obtained from all patients. The enamel sections were selected based on the following exclusion criteria: absence of cracks, hypoplasia, cusp loss, attrition, fractures, periodontal tissue remnants, and white spot lesions. After removing soft tissue residues, surface discolorations were cleaned using pumice and a brush. Each tooth was examined both macroscopically and under a light microscope (Leica M27.5, Germany) at × 30 magnification, and the eligible teeth were stored in distilled water containing 0.1% thymol solution at + 4 °C.

From the middle third of the buccal surface of each tooth, enamel specimens were sectioned using a water-cooled diamond disk (Mecatome T180, PRESI, France) and mounted in acrylic molds. The specimen surfaces were ground with 600-grit silicon carbide abrasive papers for 30 s to achieve a flat surface, and then polished with a polishing gel for 1 min (Mecatech 234, PRESI, France). After preparation, the specimens were rinsed with distilled water and stored in containers filled with distilled water at + 4 °C until further analysis.

### Formation of initial lesions

Demineralization of the specimens was achieved by immersing them in a demineralizing solution containing 2.0 mmol/L calcium, 2.0 mmol/L phosphate, and 0.075 mol/L acetate (pH 4.5) [21]. The specimens were placed in a shaking incubator (ZWYR-240, LABWIT, Australia) at 37 °C, agitated at 60 rpm, and the solutions were renewed daily to induce initial enamel lesions. After 72 h, the specimens were removed from the demineralizing solution, rinsed with distilled water, and stored in containers with distilled water at + 4 °C until post-demineralization measurements were performed [22].

### Groups and treatment protocol

The enamel specimens were randomly allocated into eight groups (*n* = 10). The experimental groups were treated with the following toothpastes, as detailed in Table 1: Group 1, experimental toothpaste containing bioactive glass (MB) (SPC Kozmetik, Türkiye); Group 2, experimental toothpaste containing bioactive glass and fluoride (MBF) (SPC Kozmetik, Türkiye); Group 3, experimental toothpaste containing protein and CaGP (MP) (SPC Kozmetik, Türkiye); Group 4, experimental toothpaste containing hydroxyapatite (MH) (SPC Kozmetik, Türkiye); Group 5, experimental toothpaste containing hydroxyapatite and fluoride (MHF) (SPC Kozmetik, Türkiye); Group 6, Sensodyne Repair & Protect (MS) (GSK, Ireland); Group 7, R.O.C.S. Sensitive (MR) (R.O.C.S. GmbH, Germany); and Group 8 (M) (control), base paste only. For this study, five new experimental toothpastes were specifically manufactured by SPC Kozmetik. Bioactive glass-based formulations were prepared using 5% bioactive glass powder with a 45S5 composition and an average particle size of 16 µm, in both fluoride-free and fluoride-containing forms. The experimental toothpaste containing protein and CaGP was formulated with hydrolyzed wheat protein and CaGP. Hydroxyapatite-based experimental toothpastes, produced in fluoride-free and fluoride-containing versions, incorporated a modified mineral structure composed of calcium carbonate coated with hydroxyapatite molecules. This modified mineral, constituting 8% of the toothpaste, consisted of 90% hydroxyapatite and 10% calcium carbonate. Unlike conventional hydroxyapatite-containing toothpastes, this formulation allowed the incorporation of fluoride without inducing precipitation of hydroxyapatite as fluorapatite within the toothpaste matrix. The experimental fluoride-enriched formulations contained 1450 ppm fluoride.

**Table 1:** Composition and classification of toothpastes applied to enamel specimens

Groups (*n* = 10) Twice a day/14 days	Content
Experimental toothpaste containing bioactive glass (MB) (SPC Kozmetik, Türkiye)	Sorbitol, Aqua, Glycerin, Hydrated Silica, Glass, Xylitol, Polysorbate 20, Cocamidopropyl Betaine, Xanthan Gum, Aroma, Phenylpropanol, Rebaudioside A, Menthol, Caprylyl Glycol, Magnesium Aspartate, Zinc Gluconate, Copper Gluconate
Experimental toothpaste containing bioactive glass and fluoride (MBF) (SPC Kozmetik, Türkiye)	Sorbitol, Aqua, Glycerin, Hydrated Silica, Glass, Xylitol, Polysorbate 20, Cocamidopropyl Betaine, Xanthan Gum, Aroma, Phenylpropanol, Rebaudioside A, Sodium Fluoride (1450 ppm), Menthol, Caprylyl Glycol, Magnesium Aspartate, Zinc, Gluconate, Copper Gluconate
Experimental toothpaste containing hydroxyapatite (MH) (SPC Kozmetik, Türkiye)	Sorbitol, Aqua, Glycerin, Hydrated Silica, Calcium Carbonate (0.8%), Hydroxyapatite (7.2%), Xylitol, Polysorbate 20, Cocamidopropyl Betaine, Xanthan Gum, Aroma, Phenylpropanol, Rebaudioside A, Caprylyl Glycol
Experimental toothpaste containing hydroxyapatite and fluoride (MHF) (SPC Kozmetik, Türkiye)	Sorbitol, Aqua, Glycerin, Hydrated Silica, Calcium Carbonate (0.8%), Hydroxyapatite (7.2%), Xylitol, Polysorbate 20, Cocamidopropyl Betaine, Xanthan Gum, Aroma, Phenylpropanol, Rebaudioside A, Caprylyl Glycol, Sodium Fluoride (1450 ppm)
Experimental toothpaste containing protein and calcium glycerophosphate (MP) (SPC Kozmetik, Türkiye)	Sorbitol, Aqua, Glycerin, Hydrated Silica, Xylitol, Polysorbate 20, Hydrolyzed Wheat Protein (2%), Cocamidopropyl Betaine, Calcium Glycerophosphate (1.25%), Xanthan Gum, Aroma, Magnesium Chloride, Phenylpropanol, Rebaudioside A, Caprylyl Glycol
Sensodyne Repair and Protect (MS) (GSK, Ireland)	Glycerin, PEG-8, Hydrated Silica, Calcium Sodium Phosphosilicate (NOVAMIN), Cocamidopropyl Betaine, Sodium Methyl Cocoyl Taurate, Aroma, Titanium Dioxide, Carbomer, Sodium Fluoride, Sodium Saccharin, Limonene
R.O.C.S. Sensitive (MR) (R.O.C.S. GmbH, Germany)	Aqua, Glycerin, Dicalcium Phosphate Dihydrate, Xylitol, Hydroxyapatite, Silica, Xanthan Gum, Aroma, Calcium Glycerophosphate, Cocamidopropyl Betaine, Sodium Lauroyl Sarcosinate, Hydroxyacetophenone, Sodium Benzoate, Sodium Saccharin, Magnesium Chloride, Sodium Methylparaben, Sodium Propylparaben, O-Cymen-5-ol, Limonene
Base Toothpaste (M) (SPC Kozmetik, Türkiye)	Sorbitol, Aqua, Glycerin, Hydrated Silica, Glass, Xylitol, Polysorbate 20, Cocamidopropyl, Betaine, Xanthan Gum, Aroma, Phenylpropanol, Rebaudioside A, Menthol, Caprylyl Glycol, Magnesium Aspartate, Zinc Gluconate, Copper Gluconate

### pH-cycling protocol

The specimens were subjected to a pH-cycling regimen using a remineralization solution [23] [(1.5 mmol/L Ca(NO_3_)_2_·H_2_O, 0.9 mmol/L NaH_2_PO_4_·H_2_O, 150 mmol/L KCl, 0.05 µg F/ml (NaF) in 0.1 mol/L Tris buffer, pH 7.0)] and a demineralization solution [21] [(2.0 mmol/L calcium, 2.0 mmol/L phosphate in 75 mmol/L acetate buffer, pH 4.5)]. For remineralization, the specimens were placed in beakers and agitated in a shaking incubator (ZWYR-240, LABWIT, Australia) at room temperature (20–22 °C) at 60 rpm.

Toothpastes assigned to each group were applied to the specimen surfaces using a toothbrush for 30 s, twice daily. Following each application, the specimens were rinsed with distilled water and subsequently immersed in the remineralization solution. Throughout the 14 day experimental period, all groups were subjected to a daily cycle consisting of 18 h of remineralization and 6 h of demineralization, as outlined in Table 2.

**Table 2:** Enamel remineralization protocol applied over 14 days

Time	Procedure
08.00–11.00	Demineralization solution
11.00–11.02	Application of the toothpastes
11.02–15.00	Remineralization solution
15.00–18.00	Demineralization solution
18.00–18.02	Application of the toothpastes
18.02–08.00	Remineralization solution

Tooth brushing was performed using the Press-on Force-Guided (PFG) brushing simulator, developed by Tağtekin and Korkut at Marmara University (Istanbul, Türkiye). This simulator was equipped with a pressure sensor, and this custom-built device was designed to simulate both electric and manual tooth brushing. It also featured an integrated drainage system that enabled the removal of excess water during brushing, thereby preventing the accumulation of toothpaste and water on the specimen surfaces [24].

**Fig. 1:**
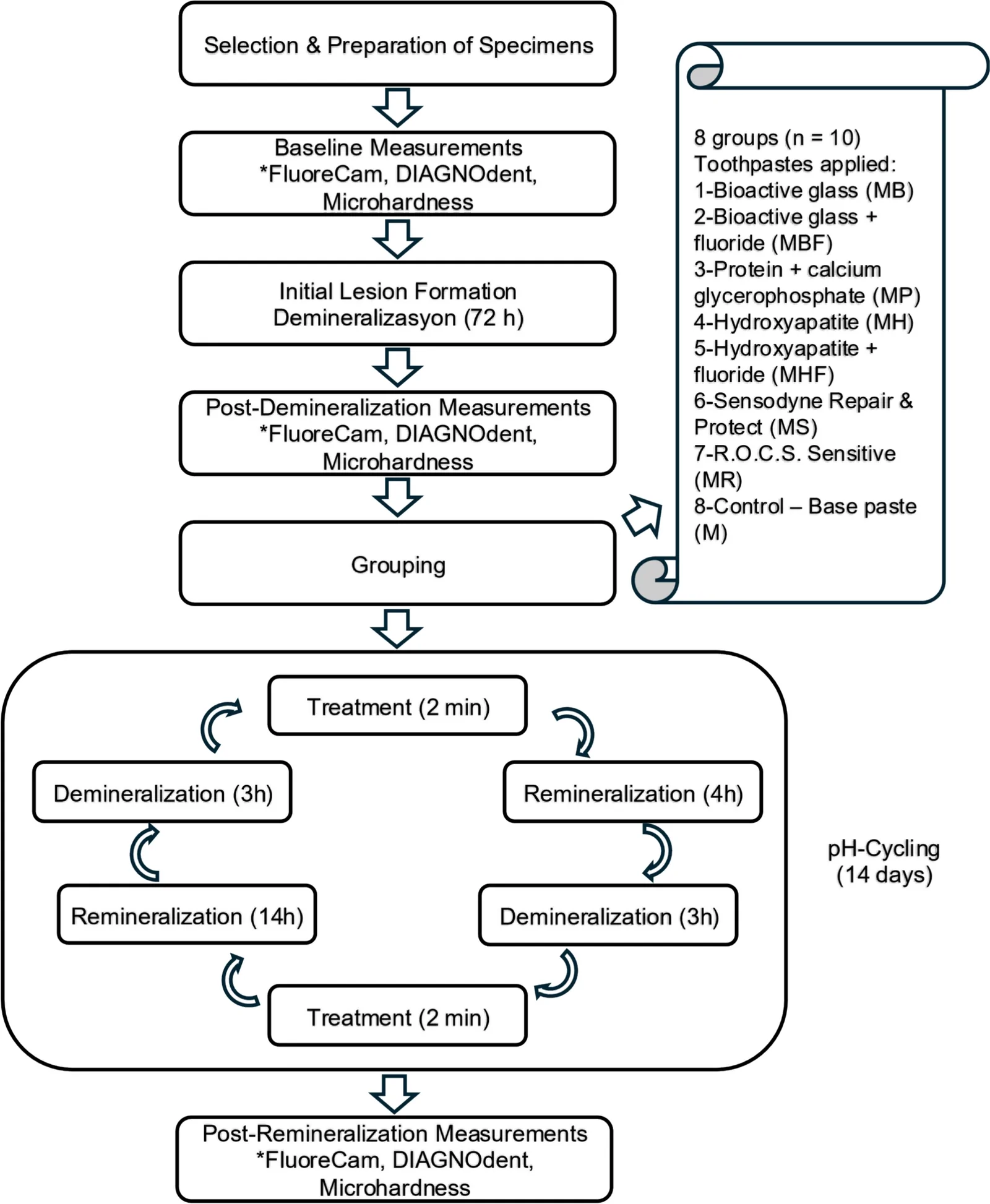
Schematic overview of the experimental procedures

Brushing procedures were performed using a rechargeable toothbrush equipped with a pressure sensor. During use, the toothbrush body was fixed in a stationary position, allowing only the brush head to move. Tooth brushing was performed using an Oral-B Sensi UltraThin brush head (Procter & Gamble, Schwalbach, Germany), which is characterized by its soft and fine bristles. For each brushing procedure, 1 g of the assigned toothpaste was used per specimen and diluted with artificial saliva at a 1:1 ratio to standardize the application. The diluted mixture was applied to the specimen surfaces and left in contact for 2 min before brushing. Brushing was then performed under constant pressure, twice daily for 30 s, with a force of 200–220 g, over a period of 14 days. A schematic overview summarizing the experimental procedures is presented in Fig. 1.

### Measurements

#### FluoreCam analysis

The FluoreCam system (Daraza Corporate Headquarters, Indiana, USA) enables the comparison of sound enamel areas with demineralized regions in terms of both size and fluorescence intensity by capturing images at different stages. In this study, fluorescence measurements of enamel specimens were performed at three stages: T1-baseline (prior to treatment), T2-post-demineralization (after artificial caries lesions were induced), and T3-post-remineralization (after completion of the 14-day remineralization protocol). Prior to each measurement, specimens were removed from moist storage containers, gently dried with a soft paper towel, and imaged using the intraoral attachment of the FluoreCam device. The captured images and measurement data were recorded in the FluoreCam software. Changes in fluorescence values, measured using the FluoreCam system, served as indirect indicators of enamel mineral density. Based on the principle that demineralized enamel exhibits lower fluorescence and remineralized areas show increased fluorescence, this method enabled the objective evaluation of structural differences between sound enamel, demineralized lesions, and post-treatment recovery through the software of the FluoreCam device.

#### DIAGNOdent measurements

To evaluate mineral changes on the enamel surface, all specimens were examined at three different time points: T1-baseline (prior to treatment), T2-post-demineralization (after artificial caries lesions were induced), and T3-post-remineralization (after completion of the 14 day remineralization protocol). Measurements were performed using the DIAGNOdent Pen device (Kavo, Biberach, Germany). During each measurement, the conical tip of the device was placed perpendicularly onto the specimen surface, and the type B probe, recommended for flat surface measurements, was used. For each specimen, readings from three different regions were recorded, and their mean value was calculated as the representative score. In accordance with the manufacturer’s recommendations, the device was calibrated with the standard ceramic reference provided by the manufacturer prior to each measurement. This standardized protocol enabled objective and comparable assessment of mineral changes occurring across different stages of the study.

In this study, the measurements obtained from the device were interpreted according to the criteria established by Lussi and Hellwig [25], and classified as follows: values between 0 and 13 indicated sound dental tissue, whereas values between 14 and 20 reflected the presence of a lesion confined to the outer enamel layer.

#### Microhardness testing

Surface microhardness measurements of enamel specimens were conducted using a microhardness tester (HMV-II, Shimadzu Corporation, Kyoto, Japan). A Vickers diamond indenter with a pyramidal geometry was employed to create indentations on the enamel surface. A constant load of 200 g (1.96 N) was applied for 15 s to each specimen.

The diagonal lengths of the indentations were measured using a × 40 objective lens, which provides a minimum resolution of 0.01 µm. Measurements were performed at three distinct stages: T1-baseline (prior to treatment), T2-post-demineralization (after artificial lesion formation), and T3-post-remineralization (following the completion of a 14-day remineralization protocol). For each specimen, three indentations were made in both the demineralized and remineralized regions. The mean of these three values was calculated to obtain the average surface microhardness for each specimen. This experimental setup allowed for a quantitative comparison of hardness changes throughout the different phases of the study.

### Statistical analysis

#### Sample size calculation

The primary outcome of the present in vitro study was the change in enamel surface microhardness after the remineralization protocol (T3–T2). An a priori specimen size calculation was performed for a one-way ANOVA with eight independent groups, using an alpha level (α) of 0.05 and a desired statistical power (1–β) of 0.80. Based on the magnitude of between-group differences reported in previous in vitro studies evaluating the remineralization efficacy of bioactive glass–containing and nano-hydroxyapatite toothpastes on enamel microhardness [26, 27], a large effect size (Cohen’s f ≈ 0.40–0.45) was considered reasonable for planning purposes. Using G*Power (version 3.1, University of Düsseldorf, Germany), the minimum required total specimen size was calculated as *N* = 78 (approximately 10 specimens per group). In the present study, a total of 80 enamel specimens (*n* = 10 per group) were included, fulfilling the required specimen size.

#### Data analysis

All statistical analyses were performed using IBM SPSS Statistics 20.0. The normality of the data distribution was assessed using the Shapiro–Wilk test. For each outcome variable (FluoreCam size, FluoreCam intensity, DIAGNOdent Pen, and Vickers microhardness), one-way ANOVA was used to compare the mean values among the eight independent groups at each time point (T1, T2, T3) and for each inter-stage difference (T2–T1, T3–T1, T3–T2). Post hoc comparisons were conducted using Tukey’s HSD test. To evaluate changes within each group across the three time points, repeated-measures ANOVA was performed. Mauchly’s test was used to assess sphericity, and Greenhouse–Geisser corrections were applied when necessary. Effect sizes for omnibus tests were reported as partial eta squared (η^2^ₚ) together with 95% confidence intervals. Pairwise comparisons were accompanied by Cohen’s d values (with 95% confidence intervals) to quantify the magnitude of clinical relevance beyond statistical significance. Statistical significance was set at *p* < 0.05. ANOVA was employed because it partitions the total variability in the data into within-group and between-group components, enabling a statistically robust comparison of mean enamel and dentin outcomes across multiple treatment groups. By evaluating whether the between-group variance exceeds the level expected from random within-group variability, ANOVA determines whether the observed remineralization differences are attributable to the experimental treatments rather than chance fluctuations.

## Results

### FluoreCam findings

The “size” and “intensity” values extracted from FluoreCam images of enamel specimens were statistically analyzed at the baseline, post-demineralization, and post-remineralization stages. The “size” parameter represents the lesion area, indicating the total surface area of the regions identified by the FluoreCam software. An increase in this value reflects lesion enlargement. The “intensity” parameter reflects mineral loss within the lesion area identified by the system and is reported as negative (–) values. A shift towards zero in this value indicates an enhancement in mineral density within the corresponding region, thereby signifying the occurrence of remineralization. FluoreCam data do not correspond to a standard physical measurement unit; instead, the results are expressed in pixel-based units provided by the software.

The FluoreCam “size” values obtained from the enamel specimens are presented in Table 3. A statistically significant difference was observed among the group means for the (T3–T2) differences (*p* < 0.001). The highest mean was observed in the M (0.81 ± 0.46), while the lowest mean was recorded in MHF (–0.92 ± 0.7). For the other groups, the values were: MP = –0.31 ± 0.31, MR = –0.34 ± 0.25, MB = –0.57 ± 0.4, MBF = –0.61 ± 0.49, MH = –0.75 ± 0.43, and MS = –0.85 ± 0.53.

**Table 3:** Comparison of ‘size’ values obtained from FluoreCam images of enamel specimens at baseline, post-demineralization, and post-remineralization

	Baseline (T1)	Demineralization (T2)	Remineralization (T3)	Difference (T2–T1)	Difference (T3-T1)	Difference (T3-T2)	Test statistic	p**
MB	1,92 ± 0,53^a^	2,31 ± 0,6^b^	1,74 ± 0,5^abB^	0,39 ± 0,14	− 0,18 ± 0,41^A^	− 0,57 ± 0,4^A^	136,184	** < 0.001**
MBF	1,82 ± 0,66^a^	2,31 ± 0,77^b^	1,7 ± 0,46^abB^	0,5 ± 0,33	− 0,12 ± 0,42^A^	− 0,61 ± 0,49^A^	95,136	** < 0.001**
MH	1,64 ± 0,83^ab^	2,2 ± 0,95^b^	1,45 ± 0,57^aB^	0,56 ± 0,39	− 0,19 ± 0,4^A^	− 0,75 ± 0,43^A^	47,629	** < 0.001**
MHF	1,53 ± 0,97	2,04 ± 1,24	1,13 ± 0,7^B^	0,51 ± 0,41	− 0,41 ± 0,4^A^	− 0,92 ± 0,7^A^	24,647	0.001
MP	1,61 ± 0,89^a^	2,35 ± 0,97^b^	2,04 ± 0,93^abA^	0,74 ± 0,22	0,43 ± 0,36^B^	− 0,31 ± 0,31^A^	46,166	** < 0.001**
MS	2,02 ± 0,68^a^	2,64 ± 0,73^b^	1,78 ± 0,87^abB^	0,61 ± 0,34	− 0,24 ± 0,41^A^	− 0,85 ± 0,53^A^	78,727	** < 0.001**
MR	1,06 ± 0,73	1,4 ± 0,83	1,06 ± 0,68^B^	0,34 ± 0,17	− 0,01 ± 0,23^A^	− 0,34 ± 0,25^A^	22,727	0.001
M	1,32 ± 0,49^a^	1,89 ± 0,46^b^	2,69 ± 0,81^cbAB^	0,57 ± 0,3	1,37 ± 0,61^A^	0,81 ± 0,46^B^	119,446	** < 0.001**
Test Statistic	1,643	1,738	4,882	1,565	17,1	12,738		
p*	0.137	0.114	** < 0.001**	0.160	** < 0.001**	** < 0.001**		
Effect size (partial η^2^ₚ)	**–**	**–**	**–**	0.13	0.62	0.55		
95% CI (η^2^ₚ)	**–**	**–**	**–**	0.10–0.33	0.48–0.74	0.43–0.70		

Experimental toothpaste containing bioactive glass (MB), experimental toothpaste containing bioactive glass and fluoride (MBF), experimental toothpaste containing protein and calcium glycerophosphate (MP), experimental toothpaste containing hydroxyapatite (MH), experimental toothpaste containing hydroxyapatite and fluoride (MHF), Sensodyne Repair & Protect (MS), R.O.C.S. Sensitive (MR), and base paste (M)

Bold values indicate statistically significant differences (*p* < 0.001)

^A–B^no significant difference between groups sharing the same uppercase letters;

^a–c^no significant difference between time points within the same group sharing the same lowercase letters; values are presented as mean ± standard deviation

*One-way analysis of variance

**repeated measures analysis of variance

When intra-group comparisons were performed, no statistically significant differences were found between baseline and post-demineralization mean values (*p* = 0.137, *p* = 0.114), indicating homogeneous distribution across groups at the start and after demineralization. However, the control group differed significantly from the treatment groups in terms of the (T3–T2) mean differences (*p* < 0.001), while no statistically significant differences were found among the treatment groups themselves. The greatest reduction in lesion size was observed in MHF (–0.92 ± 0.7) and MS (–0.85 ± 0.53), respectively.

**Fig. 2:**
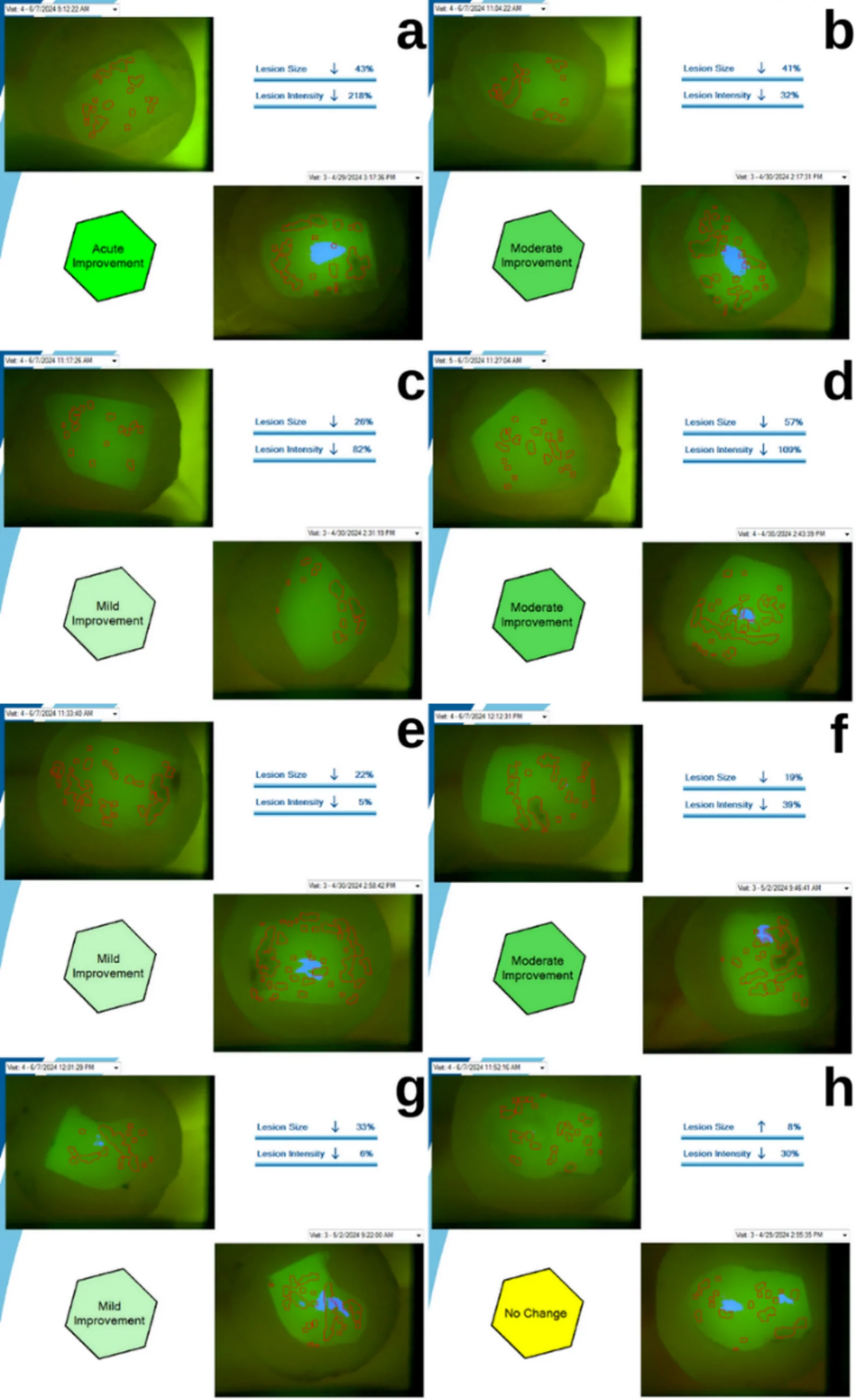
Comparison of fluorescence changes on enamel specimens between demineralization and after treatment by the FluoreCam system; **a** Experimental toothpaste containing bioactive glass; **b** Experimental toothpaste containing bioactive glass and fluoride; **c** Experimental toothpaste containing hydroxyapatite; **d** Experimental toothpaste containing hydroxyapatite and fluoride; **e** Experimental toothpaste containing protein and calcium glycerophosphate; **f** Sensodyne Repair and Protect; **g** R.O.C.S. Sensitive; **h** Control group

The FluoreCam “intensity” values obtained from enamel specimens are presented in Table 4. Considering the T3–T2 differences, the highest mean mineral gain was observed in MHF (4.22 ± 2.24), followed by MS (4.12 ± 1.57), MBF (4.02 ± 2.73), and MR (3.84 ± 2.31). The lowest mineral gain was recorded in M (1.84 ± 1.89). No statistically significant differences were found among the mean T3–T2 values between groups (p = 0.419). The fluorescence changes observed following treatment are presented in Fig. 2.

**Table 4:** Comparison of ‘intensity’ values obtained from FluoreCam images of enamel specimens at baseline, post-demineralization, and post-remineralization

	Baseline (T1)	Demineralization (T2)	Remineralization (T3)	Difference (T2–T1)	Difference (T3-T1)	Difference (T3-T2)	Test statistic	p**
MB	− 8,12 ± 2,11^ab^	− 10,59 ± 3,8^a^	− 7,87 ± 3,93^b^	− 2,47 ± 4,9	0,26 ± 4,93	2,73 ± 2,15	127,379	** < 0.001**
MBF	− 10,22 ± 2,86^ab^	− 10,75 ± 2,89^a^	− 6,73 ± 1,19^b^	− 0,53 ± 2,23	3,5 ± 3,25	4,02 ± 2,73	223,794	** < 0.001**
MH	− 9,89 ± 2,49^a^	− 10,16 ± 3,11^b^	− 7,27 ± 1,74^c^	− 0,27 ± 2,37	2,62 ± 2,59	2,89 ± 3,33	201,237	** < 0.001**
MHF	− 10,69 ± 3,08^a^	− 11,67 ± 2,98^ab^	− 7,45 ± 1,77^ca^	− 0,98 ± 0,42	3,24 ± 2,48	4,22 ± 2,24	150,468	** < 0.001**
MP	− 10 ± 2,79^ac^	− 11 ± 2,72^a^	− 7,85 ± 1,49^bc^	− 1 ± 3,11	2,15 ± 3,66	3,15 ± 3,16	387,794	** < 0.001**
MS	− 9,58 ± 3,34^ac^	− 11,15 ± 2,88^a^	− 7,03 ± 2,05^cb^	− 1,57 ± 2,01	2,55 ± 2,5	4,12 ± 1,57	119,248	** < 0.001**
MR	− 8,85 ± 2,34^ac^	− 11,16 ± 2,3^a^	− 7,32 ± 1,85^cb^	− 2,31 ± 2,43	1,53 ± 2,11	3,84 ± 2,31	250,848	** < 0.001**
M	− 8,7 ± 2,22^ab^	− 10,04 ± 1,78^a^	− 8,19 ± 2,01^cb^	− 1,33 ± 1,69	0,51 ± 1,8	1,84 ± 1,89	243,462	** < 0.001**
Test Statistic	0,943	0,326	0,456	0,777	1,364	1,028		
p*	0.479	0.940	0.863	0.608	0.234	0.419		
Effect size (partial η^2^ₚ)	**–**	**–**	**–**	0.07	0.12	0.09		
95% CI (η^2^ₚ)	**–**	**–**	**–**	0.03–0.30	0.09–0.36	0.06–0.30		

Experimental toothpaste containing bioactive glass (MB), experimental toothpaste containing bioactive glass and fluoride (MBF), experimental toothpaste containing protein and calcium glycerophosphate (MP), experimental toothpaste containing hydroxyapatite (MH), experimental toothpaste containing hydroxyapatite and fluoride (MHF), Sensodyne Repair & Protect (MS), R.O.C.S. Sensitive (MR), and base paste (M)

Bold values indicate statistically significant differences (*p* < 0.001)

^A–B^no significant difference between groups sharing the same uppercase letters

^a–c^no significant difference between time points within the same group sharing the same lowercase letters; values are presented as mean ± standard deviation

^*^One-way analysis of variance

^**^ repeated measures analysis of variance

### DIAGNOdent Pen findings

The DIAGNOdent Pen values obtained from enamel specimens are presented in Table 5. When intra-group comparisons were performed, no statistically significant differences were found between baseline and post-demineralization mean values (*p* = 0.065, *p* = 0.092), indicating homogeneous distribution across groups at these stages. A statistically significant difference was detected among the group means for the (T3–T2) differences (*p* < 0.001). The highest mean was observed in M (–3.3 ± 0.78), while the lowest mean was recorded in MB (–8 ± 1.48). The corresponding values were –7.2 ± 0.87 for MH, –7.4 ± 1.2 for MHF, –7.4 ± 0.66 for MP, –7.5 ± 1.2 for MS, –7.5 ± 1.2 for MR, and –7.8 ± 0.98 for MBF.

**Table 5:** Comparison of values obtained with the DIAGNOdent Pen from enamel specimens at baseline, post-demineralization, and post-remineralization

	Baseline (T1)	Demineralization (T2)	Remineralization (T3)	Difference (T2–T1)	Difference (T3-T1)	Difference (T3-T2)	Test statistic	p**
MB	7,4 ± 1,91^a^	15,5 ± 1,63^b^	7,5 ± 1,2^aA^	8,1 ± 2,26	0,1 ± 1,51^A^	− 8 ± 1,48^A^	609,783	** < 0.001**
MBF	6,8 ± 2,44^a^	14,7 ± 1,49^b^	6,9 ± 0,94^aA^	7,9 ± 2,21	0,1 ± 2,55^A^	− 7,8 ± 0,98^A^	489,154	** < 0.001**
MH	7 ± 2,1^a^	14,9 ± 1,3^b^	7,7 ± 1,35^aA^	7,9 ± 1,37	0,7 ± 1,42^A^	− 7,2 ± 0,87^A^	414,151	** < 0.001**
MHF	7,5 ± 1,12^a^	15,4 ± 1,28^b^	8 ± 1,1^aA^	7,9 ± 1,51	0,5 ± 0,67^A^	− 7,4 ± 1,2^A^	1062,211	** < 0.001**
MP	7,8 ± 1,47^a^	15,4 ± 0,8^b^	8 ± 0,89^aA^	7,6 ± 1,43	0,2 ± 1,25^A^	− 7,4 ± 0,66^A^	1296	** < 0.001**
MS	6,6 ± 1,11^a^	14,4 ± 0,49^b^	6,9 ± 1,14^aA^	7,8 ± 1,17	0,3 ± 1,49^A^	− 7,5 ± 1,2^A^	2129,389	** < 0.001**
MR	5,4 ± 0,8^a^	14,2 ± 0,6^b^	6,7 ± 0,9^aA^	8,8 ± 0,98	1,3 ± 0,64^A^	− 7,5 ± 1,2^A^	2385,138	** < 0.001**
M	6,4 ± 1,11^a^	14,4 ± 1,02^b^	11,1 ± 1,45^cB^	8 ± 1,84	4,7 ± 2,19^B^	− 3,3 ± 0,78^B^	2134,846	** < 0.001**
Test statistic	2,013	1,846	13,807	0,413	8,577	17,885		
p*	0.065	0.092	** < 0.001**	0.0891	** < 0.001**	** < 0.001**		
Effect size (partial η^2^ₚ)	**–**	**–**	**–**	0.04	0.45	0.63		
95% CI (η^2^ₚ)	**–**	**–**	**–**	0.04–0.25	0.29–0.64	0.51–0.77		

Experimental toothpaste containing bioactive glass (MB), experimental toothpaste containing bioactive glass and fluoride (MBF), experimental toothpaste containing protein and calcium glycerophosphate (MP), experimental toothpaste containing hydroxyapatite (MH), experimental toothpaste containing hydroxyapatite and fluoride (MHF), Sensodyne Repair & Protect (MS), R.O.C.S. Sensitive (MR), and base paste (M)

Bold values indicate statistically significant differences (*p* < 0.001)

^A–B^no significant difference between groups sharing the same uppercase letters

^a–c^no significant difference between time points within the same group sharing the same lowercase letters; values are presented as mean ± standard deviation

^*^One-way analysis of variance

^**^ repeated measures analysis of variance

In post-remineralization (T3) assessments, the control group differed significantly from all treatment groups for both (T3–T1) and (T3–T2) contrasts, indicating significantly lower mineral gain in the control group. At T3, the most favorable mean values were observed in MR (6.7 ± 0.90), MS (6.9 ± 1.14), and MBF (6.9 ± 0.94) groups; however, no statistically significant differences were detected among the treatment groups. At the T3 stage, the most favorable outcomes were observed in MR (6.7 ± 0.9), MS (6.9 ± 1.14), and MBF (6.9 ± 0.94) groups; however, no statistically significant differences were detected among the treatment groups.

### Microhardness findings

The microhardness values obtained from enamel specimens are presented in Table 6. A statistically significant difference was observed among the mean values of the (T3–T2) differences across groups (*p* < 0.001). The highest mean was found in MHF (60.65 ± 21.92), while the lowest mean was recorded in M (9.46 ± 14.28).

**Table 6:** Comparison of microhardness values obtained from enamel specimens at baseline, post-demineralization, and post-remineralization

	Baseline (T1)	Demineralization (T2)	Remineralization (T3)	Difference (T2–T1)	Difference (T3–T2)	Difference (T3–T2)	Test statistic	p**
MB	338,34 ± 22,32^a^	286,88 ± 11,84^b^	318,65 ± 22,8^abABCD^	− 51,46 ± 26,47	− 19,69 ± 29,41^AB^	31,76 ± 30,43^AB^	8153,213	** < 0.001**
MBF	351,54 ± 25,87^a^	287,85 ± 10,41^b^	332,32 ± 13,29^aABCD^	− 63,69 ± 25,4	− 19,22 ± 19,74^AB^	44,48 ± 10,3^AB^	4936,562	** < 0.001**
MH	351,55 ± 26,69^a^	293,42 ± 6,66^b^	339,18 ± 16^aABCD^	− 58,13 ± 23,54	− 12,38 ± 22,5^AB^	45,76 ± 12,74^AB^	4802,174	** < 0.001**
MHF	347,31 ± 17,12^a^	288,14 ± 6,4^b^	348,79 ± 19,51^aA^	− 59,17 ± 17,67	1,48 ± 17,75^AC^	60,65 ± 21,92^AC^	8412,126	** < 0.001**
MP	364,84 ± 17,04^a^	293,24 ± 7,69^b^	308,74 ± 15,75^cbBD^	− 71,6 ± 16,41	− 56,1 ± 25,61^B^	15,5 ± 17,23^B^	14,741,424	** < 0.001**
MS	345,62 ± 17,92^a^	293,89 ± 10,45^b^	350,96 ± 23,99^aA^	− 51,73 ± 18,93	5,34 ± 27,41^A^	57,07 ± 24,14^A^	6662,682	** < 0.001**
MR	344,37 ± 25,52^a^	295,32 ± 8,65^b^	327,47 ± 23,16^abABCD^	− 49,04 ± 23,72	− 16,9 ± 18,24^AB^	32,15 ± 20,47^AB^	3405,292	** < 0.001**
M	353,86 ± 21,46^a^	292,05 ± 8,62^b^	301,51 ± 12,16^abCD^	− 61,81 ± 24,61	− 52,35 ± 29,59^DB^	9,46 ± 14,28^DB^	22,225,785	** < 0.001**
Test Statistic	1144	1155	8188	1013	7695	7659		
p*	0.346	0.34	** < 0.001**	0.429	** < 0.001**	** < 0.001**		
Effect size (partial η^2^ₚ)	**–**	**–**	**–**	0.09	0.43	0.43		
95% CI (η^2^ₚ)	**–**	**–**	**–**	0.06–0.30	0.28–0.61	0.32–0.64		

Experimental toothpaste containing bioactive glass (MB), experimental toothpaste containing bioactive glass and fluoride (MBF), experimental toothpaste containing protein and calcium glycerophosphate (MP), experimental toothpaste containing hydroxyapatite (MH), experimental toothpaste containing hydroxyapatite and fluoride (MHF), Sensodyne Repair & Protect (MS), R.O.C.S. Sensitive (MR), and base paste (M)

^A–D^no significant difference between groups sharing the same uppercase letters

Bold values indicate statistically significant differences (*p* < 0.001)

^a–c^no significant difference between time points within the same group sharing the same lowercase letters; values are presented as mean ± standard deviation

*One-way analysis of variance

**repeated measures analysis of variance

In intra-group comparisons, both the (T3–T1) and (T3–T2) mean differences indicated that M and MHF groups differed significantly from the other groups. Based on the (T3–T2) differences, the greatest increase in microhardness was observed in MHF (60.65 ± 21.92) and MS (57.07 ± 24.14) groups, followed by MH (45.76 ± 12.74), MBF (44.48 ± 10.3), MR (32.15 ± 20.47), MB (31.76 ± 30.43), MP (15.5 ± 17.23), and M (9.46 ± 14.28). The control group exhibited significantly less microhardness recovery compared with the treatment groups, especially in comparison with MHF and MS.

When the (T3–T2) differences were examined, MBF (44.48 ± 10.3) demonstrated significantly higher mean values than MB (31.76 ± 30.43). Similarly, MHF (60.65 ± 21.92) demonstrated higher values compared with MH (45.76 ± 12.74).

## Discussion

The hypothesis of the study was accepted as, the experimental toothpastes containing bioglass, hydroxyapatite, and CaGP demonstrated significant remineralization effects on demineralized enamel surfaces, particularly when combined with fluoride. The most pronounced improvement in microhardness was recorded in the groups containing fluoride-enriched hydroxyapatite (MHF) and bioactive glass (MS). These findings underscore the pivotal role of fluoride in promoting remineralization and suggest that biomimetic agents, when co-applied with fluoride, may exert synergistic effects that further enhance enamel repair. Similarly, Farooq et al. [26] reported that a fluoride-containing bioactive glass toothpaste achieved higher enamel surface microhardness and more pronounced remineralization than fluoride toothpaste alone, as evidenced by microhardness and micro-CT analyses. These findings suggest that the synergy between fluoride and bioactive glass may be associated with optimized ion release dynamics and calcium-phosphate balance. In the present study, the addition of fluoride, particularly in formulations based on bioglass and hydroxyapatite, enhanced remineralization, as confirmed by both microhardness measurements and FluoreCam analyses. Collectively, these results support the hypothesis that combining fluoride with bioactive or biomimetic materials provides superior remineralization outcomes compared to single-agent applications.

A study conducted by Poopirom, Yimcharoen, and Rirattanapong [28] compared a fluoride-containing bioactive glass toothpaste with a conventional toothpaste containing 1500 ppm sodium fluoride on primary enamel, reporting similar increases in microhardness for both groups. Notably, despite the fluoride-containing bioactive glass toothpaste having only 530 ppm fluoride, a comparable remineralization effect was achieved. This may be explained by the bioactive glass facilitating the formation of hydroxyapatite-like crystal nuclei through the interaction of fluoride ions with free calcium and phosphate ions, thereby promoting effective mineral reorganization even at lower fluoride concentrations.

On the other hand, a study by Körner et al. [29] reported that fluoride alone produced higher remineralization than formulations containing bioactive glass or hydroxyapatite. This could be attributed to the ability of high fluoride concentrations to directly promote fluorapatite formation. When considered collectively, these three studies suggest that variations in results may stem from differences in tooth type (primary or permanent), fluoride concentration, pH conditions, and experimental protocols. Overall, it can be concluded that the combination of fluoride and bioactive glass has strong remineralization potential in both primary and permanent enamel tissues; however, its efficacy may vary depending on the fluoride concentration, pH environment, particle characteristics, and the morphological features of the enamel substrate.

In the study conducted by Güçlü et al. [30], it was reported that hydroxyapatite exhibited limited remineralization capability under neutral pH conditions, yet provided surface protection and partial subsurface repair in acidic environments through dissolution and subsequent release of calcium and phosphate ions. In our study, the significant increase in microhardness observed under a pH-cycling model that incorporated periods of acid challenge for the fluoride-stabilized hydroxyapatite formulation is consistent with these findings in the literature. Nevertheless, the high level of remineralization efficacy obtained in our research may be attributable not only to the ambient pH, but also to the modified particle structure of the hydroxyapatite used and its synergistic interaction with fluoride. The same study noted that bioactive glass promoted subsurface mineral gain under neutral conditions but predominantly formed a superficial mineral layer under acidic conditions. In contrast, in our study, the fluoride-containing bioactive glass formulations contributed significantly to the remineralization process, as demonstrated by the results of the Vickers microhardness test and the DIAGNOdent Pen; notably, the DIAGNOdent Pen findings suggested that this effect may extend into subsurface regions.

Nevertheless, as this study evaluated only surface microhardness, future investigations incorporating cross-sectional microhardness analyses are warranted to obtain more direct insights into subsurface mineralization dynamics. This method is particularly valuable for formulations containing 45S5 bioactive glass, owing to its sensitivity in detecting mineral gain within subsurface lesions. [31]. Moreover, the use of this analytical approach will allow a clearer distinction between superficial mineral precipitation and true remineralization, thereby enabling a more reliable and objective evaluation of the effectiveness of the applied treatment protocols.

Amaechi et al. reported that mouthrinses containing wheat protein facilitate the repair of eroded tooth surfaces by forming a protective layer composed of crystalline deposits [18]. In contrast to that study, we incorporated wheat protein into a toothpaste formulation and additionally included calcium glycerophosphate (CaGP) with the aim of enhancing its remineralization efficacy. The findings demonstrated that the experimental wheat-protein-containing toothpaste supported mineral gain on demineralized enamel surfaces and significantly increased microhardness. However, these improvements were observed to be more limited compared with those achieved by commercial toothpastes containing bioactive glass and hydroxyapatite. This outcome may be related to the mechanism of wheat protein, which binds calcium and phosphate ions present in saliva, transports them to the enamel surface, and facilitates mineral retention by forming a protective film layer. Moreover, the fact that the study was conducted under in vitro conditions and in an artificial saliva environment may not fully reflect the effects of these protein-based agents within the real biological milieu of the oral cavity. Hence, it is plausible that wheat-protein-based formulations could demonstrate more substantial remineralization potential under real clinical conditions.

The contribution of CaGP included in the formulation is also noteworthy in this context. The literature indicates that CaGP is an effective agent in supporting remineralization and that it can exert a synergistic effect, particularly when used in combination with fluoride [32, 33]. In addition, several studies have demonstrated that CaGP is effective as an independent remineralizing agent even in fluoride-free systems [34–36]. In the present study, when the experimental CaGP-containing toothpaste (MP) was compared with a commercial CaGP-containing product (R.O.C.S. Sensitive—MR), both formulations were found to provide a similar degree of remineralization. These findings suggest that CaGP is an effective remineralizing agent on its own; however, its remineralization potential may be further enhanced when combined with fluoride. Consequently, future studies focusing on next-generation formulations in which agents such as CaGP and wheat protein are combined with fluoride and evaluated under clinical conditions may have the potential to improve both the efficacy and the biocompatibility of remineralization.

Xylitol is an anticariogenic agent that not only suppresses acid production by oral bacteria but also functions as a calcium carrier during the remineralization process. This property is attributed to its ability to increase the bioavailability of calcium ions in saliva and facilitate their transport to the enamel surface. In contrast, sorbitol is a sugar alcohol primarily used for its humectant properties and exhibits relatively weak anticariogenic effects [37–39]. In the present study, both the experimental formulations and the base paste used in the control group contained xylitol and sorbitol. Therefore, the evaluation of remineralization efficacy primarily focused on the active agents—hydroxyapatite, bioactive glass, and protein–CaGP. This experimental design enabled a more precise and isolated assessment of the remineralization potential of the investigated active agents.

In this study, the remineralization of early enamel lesions was evaluated using a combination of microhardness testing, DIAGNOdent Pen, and FluoreCam imaging. These methods are commonly employed in the literature as complementary tools that assess different aspects of enamel mineralization [40–42]. Microhardness testing provides a quantitative assessment of the mechanical resistance of the enamel surface, directly reflecting the extent of physical remineralization [43, 44]. The DIAGNOdent device is capable of detecting bacterial byproducts trapped within subsurface porosity regions of enamel, enabling it to generate fluorescence signals even in the absence of active microbial presence. Owing to this mechanism, the device can provide reliable and meaningful data regarding demineralization and remineralization dynamics, even under bacteria-free in vitro conditions. Numerous studies have demonstrated the successful use of DIAGNOdent in assessing early enamel lesions and monitoring the effects of remineralizing agents [42, 45–47]. FluoreCam, on the other hand, offers wide-field digital fluorescence imaging and generates color-coded maps of mineral distribution, providing visually rich and clinically interpretable data [41, 48]. The combination of these three techniques allows for a more comprehensive and cross-validated evaluation of remineralization by integrating both mechanical and optical parameters [40–42].

This study has several limitations. The research was conducted entirely under in vitro conditions. Although the in vitro pH-cycling model simulates oral conditions, it cannot fully replicate factors such as salivary flow, pellicle renewal, or pH fluctuations. Therefore, the findings may not fully reflect the complexity of in vivo oral conditions. The use of artificial saliva may also have limited the effectiveness of protein-based formulations, particularly those relying on the binding and transport of calcium and phosphate ions. In addition, remineralization was assessed solely through microhardness, DIAGNOdent Pen, and FluoreCam measurements; the absence of advanced characterization techniques restricted detailed evaluation of the microstructural properties of the newly formed mineral layer. Moreover, although the required sample size was achieved, some groups exhibited considerable within-group variability, with standard deviations reaching 30–50% of the mean. This variability likely contributed to the widened confidence intervals and may explain why certain comparisons did not achieve statistical significance despite favorable trends. Future studies should, therefore, consider larger sample sizes or more rigorous specimen-standardization procedures to reduce variability and enhance the reliability and statistical power of the findings. Considering these limitations, while the results of the present study are clinically promising, in situ and in vivo research is needed before direct clinical extrapolation can be made.

The results of this in vitro study highlight the effectiveness of different remineralization agents in managing early-stage enamel demineralization. These findings may guide clinicians in selecting appropriate non-invasive treatment strategies, particularly when aiming to prevent lesion progression and promote enamel repair using bioactive and fluoride-based formulations.

## Conclusion and recommendations

The findings of this study demonstrate that toothpastes play a significant role in the remineralization of initial enamel caries. In particular, it has been demonstrated that biocompatible and functional agents such as bioactive glass, hydroxyapatite, and wheat protein–CaGP contribute to the remineralization process when incorporated into both experimental and commercial toothpastes.

## Data Availability

The datasets generated during and/or analyzed during the current study are available from the corresponding author on reasonable request.
